# Improved Detection of Capillaries in High‐Resolution Handheld Vital Microscopy by Use of the MicroTools Advanced Computer Vision Algorithm

**DOI:** 10.1111/micc.70045

**Published:** 2026-01-04

**Authors:** Philippe Guerci, Can Ince, Olcay Dilken, Thibaut Belveyre, Coline Lapoix, Jonathan Montomoli, Matthias P. Hilty

**Affiliations:** ^1^ Department of Anesthesiology and Critical Care Medicine University Hospital of Nancy Nancy France; ^2^ INSERM U111 University of Lorraine Nancy France; ^3^ Department of Intensive Care Erasmus MC, University Medical Center Rotterdam the Netherlands; ^4^ Active Medical BV Leiden the Netherlands; ^5^ Department of Anaesthesia and Critical Care Magellan Medico‐Surgical Center, CHU Bordeaux Bordeaux France; ^6^ Infermi Hospital Department of Anesthesia and Intensive Care Rimini Italy; ^7^ University Hospital of Zurich Institute of Intensive Care Medicine Zurich Switzerland

**Keywords:** computer vision, handheld vital microscopy, hemodynamic monitoring, microcirculation, MicroTools

## Abstract

**Objective:**

Handheld vital microscopy (HVM) enables bedside visualization of the microcirculation, with major improvements from sidestream darkfield (SDF) to incident dark field (IDF) imaging. Although IDF offers high‐resolution images, standard analysis methods require down‐sampling to match SDF's lower field of view (FOV) and pixel density. MicroTools, an automated stabilization and analysis algorithm, has previously been validated for use with SDF image sequences. This study aimed to assess the accuracy and precision of microcirculatory analysis using MicroTools on full‐frame, high‐resolution IDF images.

**Methods:**

Image sequences from a previous study were re‐analyzed in both IDF and down‐sampled SDF formats. Microcirculatory parameters—including total vessel density (TVD), functional capillary density, and red blood cell velocity (RBCv)—were compared between formats. MicroTools' new stabilization algorithm was also evaluated against existing algorithms.

**Results:**

Full‐frame IDF analysis increased the FOV by 200% and pixel density by 66%, enhancing capillary detection and TVD measurements. RBCv values were lower with IDF images. The updated stabilization algorithm showed performance comparable to prior methods.

**Conclusions:**

MicroTools analysis of full‐frame IDF images improved measurement accuracy and vessel detection, with excellent agreement to standard software. Its new stabilization algorithm proved equally robust, supporting broader adoption in clinical research.

**Abbreviations**
FCD: functional capillary density
FOV: field of view
HVM: handheld intravital microscopy
IDF: incident dark field
OPS: orthogonal polarized spectral
PPV: proportion of perfused vessels
PVD: perfused vessel density
RBCv: red blood cells velocity
SDF: sidestream dark field
TVD: total vessel density

## 1 Introduction

A healthy microcirculation is a core element for maintaining the balance between oxygen delivery to tissue and oxygen consumption by the cells, to sustain organ function. In critical conditions, however, the microcirculation can be severely jeopardized, leading to organ dysfunction. Over the past two decades, advances in optical technologies for handheld vital microscopy (HVM)—including orthogonal polarized spectral (OPS) imaging [1], sidestream dark field (SDF) imaging [2], or more recently incident dark field (IDF) illumination [3], have enabled bedside visualization of the microcirculation. Numerous studies have highlighted that persistent microcirculatory disturbances, despite apparently well‐conducted resuscitation and restored macrohemodynamics, were associated with poor outcomes in critically ill patients [4, 5]. This underscores the need to monitor the microcirculation during resuscitation and consider microcirculatory parameters as potential therapeutic targets [4].

Targeting microcirculatory function represents a promising approach to hemodynamic management in critically ill and perioperative patients, provided that it can be reliably quantified at the bedside. However, several technical challenges remain. Although microscopy hardware has advanced significantly, with modern microscopes capable of capturing high‐resolution images (as seen with IDF imaging), current gold‐standard image analysis still relies on down‐sampling to match the lower resolution and field of view of earlier SDF microscopes [6]. Down‐sampling refers to the process of reducing the spatial resolution of an image by combining adjacent pixels, here to match the SDF optical geometry and resolution. Consequently, the additional information provided by the enhanced field of view and pixel density in IDF imaging remains largely untapped. Recently, an automated analysis algorithm and software called MicroTools [7] has been developed, demonstrating the ability to reproduce standard manual analysis results for SDF image sequences within a large multicenter database of critically ill and perioperative patients [8]. Notably, this algorithm also enables the analysis of full‐resolution IDF image sequences, bridging the gap between modern HVM capabilities and image analysis.

However, automated analysis of HVM image sequences, particularly those utilizing mean image generation [7] is more dependent on image sequence stabilization than manual methods. A computationally efficient and fully integrated stabilization algorithm is therefore essential for the practical application of automated analysis in both research and clinical settings. To address this, a stabilization algorithm has been implemented within the MicroTools software to make HVM image sequence stabilization independent of microscope hardware or proprietary software.

The hypotheses of the present study were twofold: (i) analyzing HVM image sequences in the full‐resolution IDF format would improve the detection of capillary total vessel density (TVD) compared to down‐sampled SDF‐format sequences, and (ii) a newly developed stabilization algorithm for HVM image sequences would produce results equivalent to those obtained using existing stabilization methods. To test these hypotheses, we compared automated analysis results from HVM image sequences in the IDF format with results from the same sequences cropped and down‐sampled to the SDF format. Additionally, we evaluated the performance of the new stabilization algorithm against two commonly used algorithms in the research setting.

## 2 Materials and Methods

### 2.1 Study Population and Design

In the present study, HVM image sequences acquired in a previous study [7] were re‐analyzed using the MicroTools advanced computer vision algorithm. The original study involved 41 healthy human volunteers (age 46 ± 2 years, 22/41 (54%) male, weight 69.0 ± 1.8 kg, height 174 ± 1 cm, body mass index 23.1 ± 0.8 kg.m^−2^) at the University Hospital of Bern, Switzerland. The sublingual microcirculation was examined at baseline and 30 s after the topical application of acetylcholine and nitroglycerin solutions to assess microvascular reactivity using a CytoCam [7]. Microcirculatory imaging was performed using a Cytocam incident dark‐field microscope (Braedius Medical, Huizen, The Netherlands), linked to a laptop through a dedicated camera control unit. Video sequences were captured at 25 frames per second with a spatial resolution of 2208 × 1648 pixels, corresponding to a field of view of 1.78 mm^2^. Each sequence lasted 10 s. All recordings were obtained by the same trained investigator, with subjects examined in the supine position and at rest. For each participant, four clips of randomly selected sublingual sites were acquired. In addition, two recordings were performed following topical administration of acetylcholine and two after nitroglycerin (detailed thereafter), to assess endothelial‐dependent and endothelial‐independent microvascular reactivity, respectively.

In the underlying study, microvascular reactivity was introduced as a measure of the maximum recruitable capillary density and red blood cell flow resulting from nitric oxide release. This release was triggered by endothelial cell stimulation with acetylcholine and enzymatic transformation of nitroglycerin, respectively [9]. The dataset from this study provides a wide range of vascular densities, making it well suited for evaluating the performance of the MicroTools algorithm.

To induce microvascular changes, 500 μg of acetylcholine (3.4 μmol, prepared as 0.05 mL of 1% (6.8 × 10^−2^ M) acetylcholine solution) was applied to the sublingual area immediately after reconstituting acetylcholine lyophilizate (Miochol E, Bausch & Lomb Swiss, Zug, Switzerland). This was followed by 5 μg of nitroglycerin (2.2 × 10^−2^ μmol, prepared as 0.05 mL of 1% (4.4 × 10^−2^ M) nitroglycerin solution (Perlinganit isotonic infusion solution, UBC Pharma, Bulle, Switzerland), diluted 1:102 with 0.9% sodium chloride). Both interventions were designed to affect the local microcirculation consistently without altering systemic circulation [9].

The study was conducted with approval from the institutional Ethics Board of the University of Bern (KEK 226/12) and registered under http://clinicaltrials.gov (identifier NCT01953198). Written informed consent was obtained from all participants.

All HVM image sequences were recorded with a Cytocam in IDF format and subsequently stabilized and analyzed using MicroTools. Analyses were performed in two formats: the original IDF image format and an SDF image format created by down‐sampling the IDF recordings. For comparison of stabilization methods, the original IDF recordings were converted to SDF format and stabilized using AVA 3.2 and MicroTools algorithms. These stabilized sequences were analyzed using MicroTools and compared with sequences stabilized using CCTools 1.7.12. After stabilization, the image sequences were converted to SDF format, which reduced the field of view.

Image quality was subjectively evaluated using the microcirculation image quality score (MIQS), which rates six domains: illumination, recording duration, focus, content, stability, and applied pressure prior to analysis with MicroTools for all sequences [10]. The video clips not reaching sufficient quality were discarded. This approach enabled a direct comparison of the stabilization methods and their impact on the accuracy of automated image analysis.

### 2.2 Field of View, Pixel Density, and Their Relation to the Image Analysis Algorithm

Resolution is commonly used to describe the number of pixels that constitute an image [7] However, in the context of HVM, it is often used interchangeably with the distance in the tissue represented by the center‐to‐center distance between two adjacent pixels, which is more accurately referred to as *pixel pitch* [7]. In this study, we distinguished between two related concepts: the *field of view*, which refers to the rectangular area of tissue projected onto the microscope's sensor, and the *pixel density*, defined as the ratio between the distance in the tissue and the number of pixels corresponding to that distance. The original MicroTools algorithm [7] is inherently dependent on both pixel density and the field of view, which is determined by the number of pixels in the horizontal and vertical dimensions of an image. It was initially calibrated to analyze HVM image sequences with the field of view and pixel density specific to the SDF technique, as these properties aligned with the then‐current gold standard for HVM analysis: manual analysis using the AVA 3.2 software.

For this study, the MicroTools algorithm was extended to accommodate changes in both the field of view and pixel density of HVM image sequences, enabling analysis of the full frame of IDF image format sequences. This enhancement allows MicroTools to leverage the higher resolution and larger field of view provided by modern HVM imaging technologies.

### 2.3 Analysis Software Packages & Image Sequence Stabilization

Three software packages were used in this study. AVA 3.2 is a commercial program specifically developed for the manual analysis of SDF‐format videos, with a stabilization process optimized for manual vessel tracing. CCTools 1.7.12 is another commercial software primarily intended for manual analysis of IDF‐format videos, offering optional export to SDF resolution; its stabilization function is hardware‐dependent. Finally, MicroTools is an open‐source, fully automated analysis software that incorporates its own stabilization algorithm optimized for computational vessel detection and red blood cell velocity estimation, and operates independently of microscope hardware.

The image sequence stabilization algorithm implemented in MicroTools is based on the calculation of optical flow for a sparse feature set using the iterative Lucas–Kanade method with pyramids, as described in detail elsewhere [11, 12]. In summary, tracking features are selected using a scoring function that prioritizes corners in the λ1‐λ2 space, where λ1 and λ2 are the Eigenvalues of a matrix computed from image derivatives. These features have been shown to be highly sensitive to motion changes, regardless of direction [12]. Optical flow between frames is then iteratively calculated for these tracking features, progressing through increasingly higher resolutions [11]. To suppress the conflicting motion of individual red blood cells in HVM image sequences, a smoothing algorithm is applied across the entire span of frames. This algorithm assumes that the random motion of red blood cells across the field of view will largely cancel each other out. As a result, all motion is eliminated relative to the first frame, achieving a stabilized sequence.

### 2.4 Measurements

Standard microcirculatory parameters, described in detail elsewhere, were measured [4, 13]. Briefly:

- Total Vessel Density (TVD): This represents the total length of all capillaries containing red blood cells (both moving and stationary) divided by the field of view (FOV) and is expressed in mm/mm^2^.
- Functional Capillary Density (FCD) represents the total length of capillaries containing moving red blood cells, divided by the FOV, also expressed in mm/mm^2^.
- Percentage of Perfused Vessels (PPV) is a weighted mean (by capillary segment length) of the categorical “nonperfused” property, determined from a spatiotemporal diagram ridge velocity frequency histogram. It quantifies the proportion of vessels surpassing a defined velocity threshold.
- Red Blood Cell Velocity (RBCv): a newer parameter, RBCv, measures the weighted mean (by capillary segment length) of the absolute red blood cell velocity across all capillary segments within the FOV. It is expressed in μm/s.

### 2.5 Statistical Analysis

TVD, FCD, and RBCv values obtained from algorithmic image sequence analysis in IDF and SDF image formats, as well as different stabilization algorithms, were compared using Pearson's product–moment correlation coefficient for linear correlation and Bland–Altman analysis [14] including percentage error analysis [15].

Image properties such as field of view, pixel density, and pixel count, along with microcirculatory parameters derived from algorithmic analyses of IDF and SDF image sequences under various stabilization algorithms, were compared using linear mixed model analysis [16]. File format and stabilization method were included as fixed effects in the model, while individual intercepts for discrete image sequences and per‐sequence random slopes (representing the effect on the dependent variable) were included as random effects. Statistical significance was determined using a likelihood ratio test, comparing the full model (including the effect in question) with a null model that excluded the effect [17]. A two‐sided *p*‐value < 0.05 was considered statistically significant.

All statistical analyses were conducted using a fully scripted data management workflow within the R environment for statistical computing (version 3.4.2) [18]. Linear mixed‐effects modeling was performed using the lme4 library (version 1.1.13) [16], and graphical outputs were generated with the ggplot2 library (version 2.2.1) [19]. Results are presented as mean ± standard deviation (SD).

## 3 Results

The main characteristics of HVM devices and the different file formats used in SDF and IDF imaging are summarized in Table 1. The image sequences acquired with the Cytocam‐IDF in their native format, at 2208 × 1648 px, have a pixel density of 0.7 μm·px^−1^, compared to 1.2 μm·px^−1^ for the original SDF format obtained with the Microscan‐SDF. The Cytocam‐IDF images were spatially down‐sampled to 716 × 572 px by bilinear interpolation after cropping the field of view to match SDF optical geometry, resolution and field of view (no temporal down‐sampling was applied), resulting in a substantial loss of data. This conversion reduces the FOV by approximately one‐third when high‐resolution IDF‐format files are converted to SDF format. A total of 328 video clips were acquired in Cytocam‐IDF format of which 324 were stabilized with CCTools and 286 converted by CCTools to SDF format. The complete digital image processing workflow is presented in the Supporting Information (Figure S1).

**TABLE 1:** Comparison of file formats currently used for handheld vital microscopy.

Description	HVM recording device	File format and resolution	Technical description	Frame rate per second	Approximate field of view	Approximate pixel density
SDF image sequence	Microscan SDF Analogue (MS‐A)	avi, 716 × 572 px	AVA 3. × native recording format	25/30	0.5898 mm^2^	1.2 μm px^−1^
SDF image sequence	Microscan USB3 (MS‐U)	avi 1280 × 960 px	AVA 4. × native recording format	8–54	0.705 mm^2^	0.7 μm px^−1^
SDF image sequence	MicroSee V100/200	avi, 656 × 492 px	AVA 3. × native recording format	25	730 × 550 μm^2^	1.1 μm px^−1^
IDF image sequence, down‐sampled and cropped to SDF resolution and field‐of‐view	Cytocam IDF	avi, 716 × 572 px	CCtools AVA 3. × export	25	0.5898 mm^2^	1.2 μm px^−1^
High resolution and field‐of‐view image sequence	Cytocam IDF	mha, 2208 × 1648 px	CCtools 1.7. × native recording format	25	1.7830 mm^2^	0.7 μm px^−1^

Abbreviations: HVM, handheld vital microscopy; IDF, incident dark field; px, pixel; SDF, sidestream dark field.

### 3.1 Comparisons Between Analysis in Different FOV and Pixel Density

Comparisons of image properties and microcirculatory parameter analyses between SDF and IDF image formats are summarized in Table 2. As anticipated, the FOV was significantly larger in the IDF image format compared to the SDF image format (*p* < 0.0001), with an approximately 200% increase. Additionally, the pixel density was 66% higher in the IDF format (*p* < 0.0001). The larger FOV also led to a smaller standard deviation and improved accuracy in microcirculatory parameter measurements. The increased pixel density allowed for the detection of approximately 10% more capillaries (18.0 ± 5.4 vs. 19.5 ± 5.1 mm.mm^−2^, *p* < 0.0001). A comparable increase was observed in the TVD of larger vessels (venules), rising from 7.2 ± 1.9 to 7.7 ± 1.5 mm.mm^−2^, *p* < 0.0001.

**TABLE 2:** Functional parameters of the sublingual microcirculation as measured in image sequences recorded with an IDF microscope in healthy human volunteers and analyzed in the full field of view and resolution, and after down‐sampling and cropping to SDF field of view and resolution.

	SDF format	IDF format	*p*_format_	*p*_corr_	*r*	Upper LOA	Lower LOA	Bias = mean difference between paired measurements	Precision = standard deviation of bias	Percentage error = (1.96 × precision/mean of reference) × 100
	*n* = 242	*n* = 323								
Image properties										
Field of view [mm^2^]	0.59 ± 0.01	1.50 ± 0.16	< 0.0001							
Pixel density [μm px^−1^]	717 ± 5	2047 ± 114	< 0.0001							
Horizontal pixels [px]	571 ± 11	1497 ± 107	< 0.0001							
Vertical pixels [px]	0.71 ± 0.00	1.21 ± 0.00	< 0.0001							
Microcirculatory parameters										
Small vessels (capillary) TVD [mm mm^−2^]	18.0 ± 5.4	19.5 ± 5.1	< 0.0001	< 0.0001	0.84	7.4	−4.4	1.5	3.0	12.4
FCD [mm mm^−2^]	16.8 ± 5.2	18.6 ± 4.9	< 0.0001	< 0.0001	0.83	7.5	−4.0	1.8	2.9	15.0
PPV [%]	94 ± 8	96 ± 5	< 0.0001	< 0.0001	0.79	0.1	−0.1	0.0	0.0	2.4
RBCv [μm s^−1^]	336 ± 73	327 ± 56	< 0.0001	< 0.0001	0.88	57.7	−83.1	−12.7	35.2	−2.3
Large vessels TVD [mm mm^−2^]	7.2 ± 1.9	7.7 ± 1.5	< 0.0001	< 0.0001	0.48	4.0	−3.0	0.5	1.7	14.4

*Note:* The image sequences were stabilized using MicroTools. Values are given as mean ± SD.

Abbreviations: FCD, functional vessel density; LOA, level of agreement; PPV, proportion of perfused vessels; RBCv, red blood cell velocity; TVD, total vessel density.

Correlations between the SDF and IDF image formats for TVD, FCD, PPV, and RBCv were strong (*p* < 0.0001). However, Bland–Altman analysis revealed a consistent bias, emphasizing that more capillaries were detected in the IDF image format. The increase in capillary detection was proportional to the capillary density present in the lower‐resolution SDF format. Pearson correlation indicated that most of the variability in the IDF image format could be attributed to variability within the SDF format. Overall, the limits of agreement between SDF and IDF image formats were acceptable, with minimal bias except for RBCv (Table 2).

**FIGURE 1:**
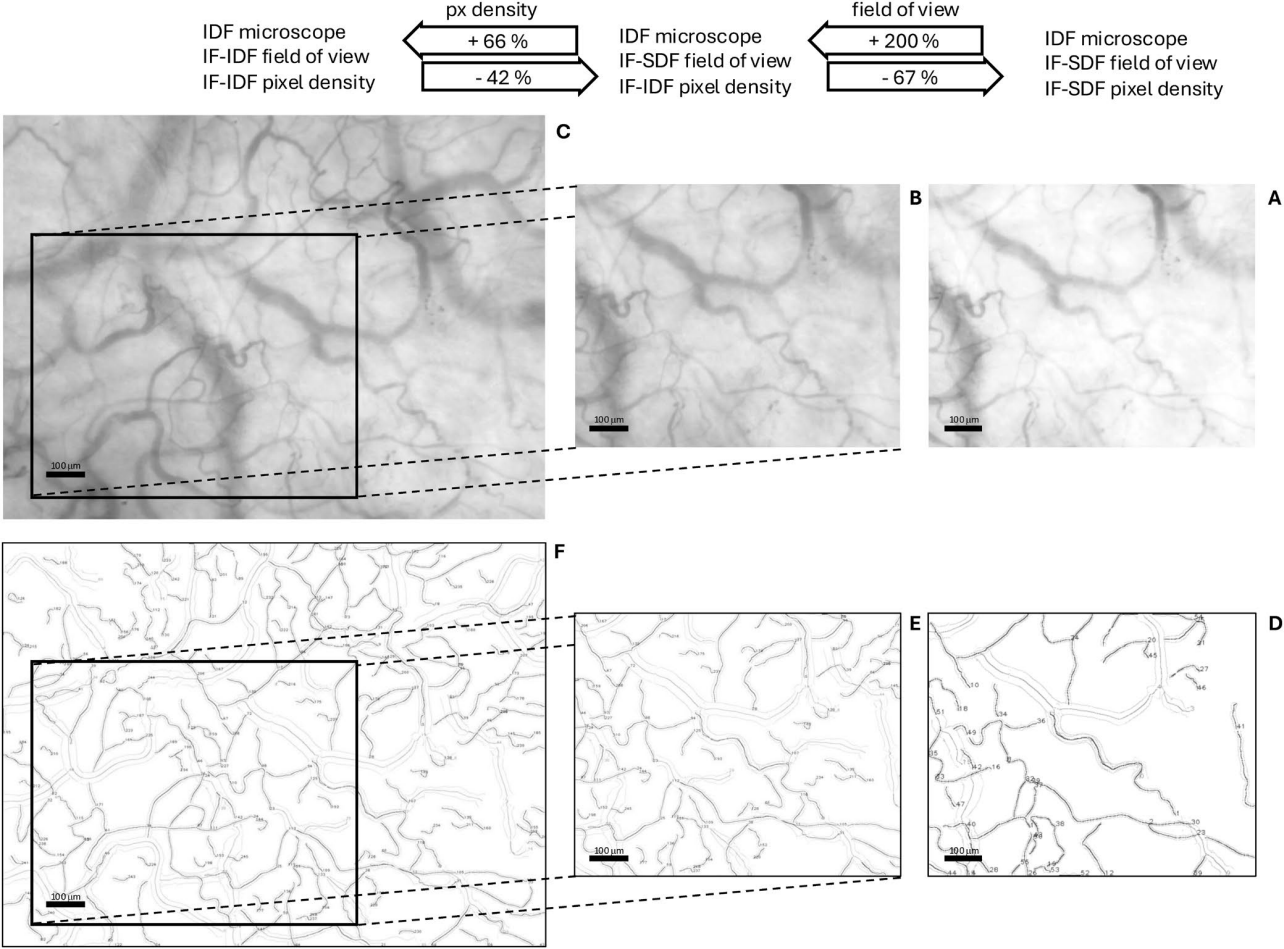
Representative examples of mean images and detectable microvessels recorded by an IDF microscope. This shows a 10% increase in detectable capillary TVD depending on file format used during the analysis stage. After cropping and down‐sampling, SDF format corresponds to the current “gold standard” for HVM image analysis (A and D). The original, 66% higher pixel density supported by the IDF microscope are shown in (B and E), the additional 200% increase in field of view corresponding to the original IF‐IDF format provided by the microscope in (C and F). Combined with the previous finding of a 30% increase in detectable capillary TVD when comparing SDF image sequence recorded by a current generation versus a previous generation microscope [3], an increase in detectable capillary TVD of 40%–50% may be expected between the two last generations of HVM microscopes.

Figure 1 depicts the actual increase in FOV and pixel density between SDF and IDF image format sequence clips. While the SDF image format (Panel A, Figure 1) appears brighter with lower contrast compared to the IDF format, both panels represent the same image sequence clip initially acquired with an IDF device. The image was subsequently cropped and down‐sampled for the SDF format, but brightness and contrast settings were left unaltered. This figure underscores the significance of preserving the high resolution of the original IDF‐acquired images.

**FIGURE 2:**
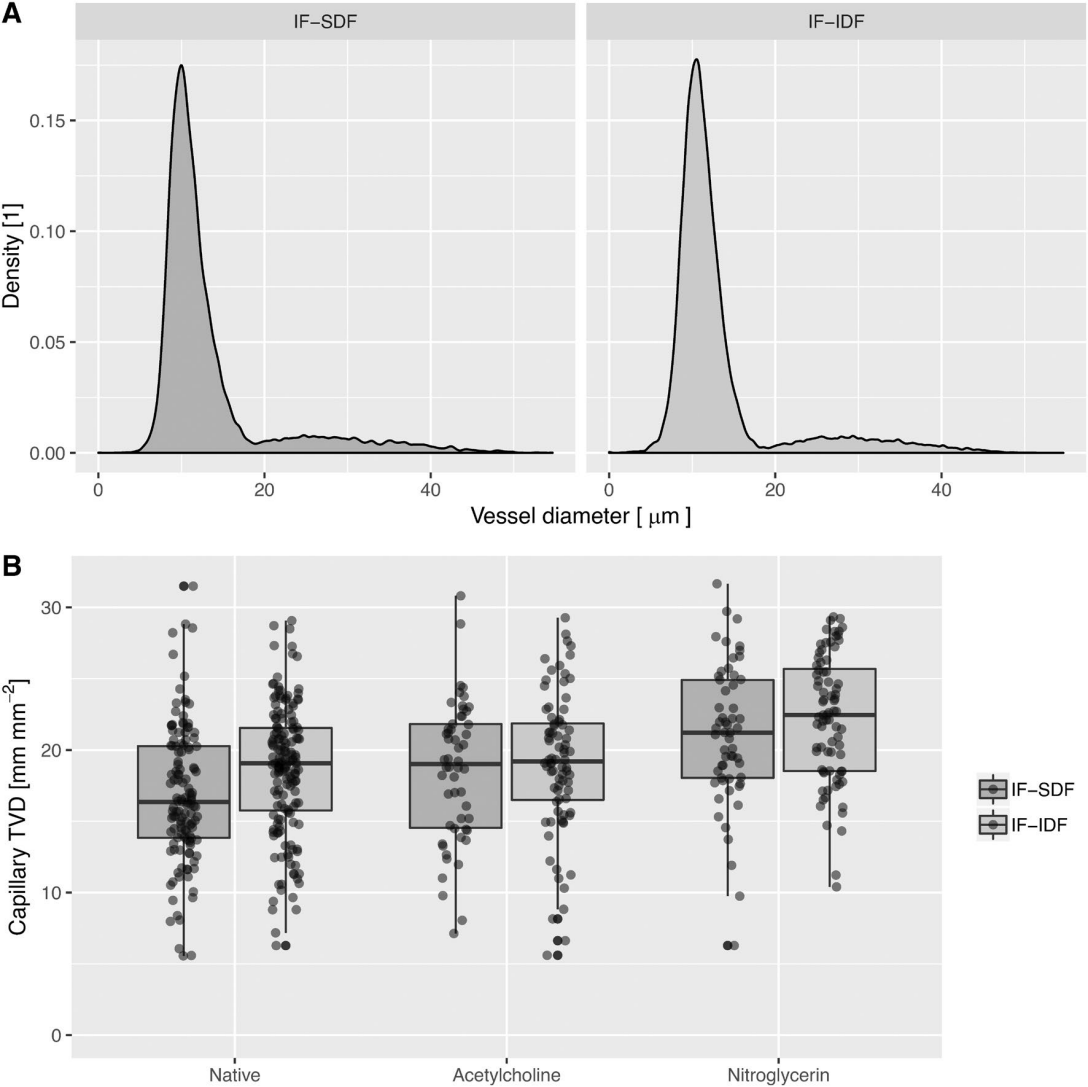
Vessel diameter density distributions are similar in the analysis of SDF and IDF image format sequences. (A) A higher capillary TVD is detectable in analysis of IDF image format sequences, while both methods differentiate well between the topical application of acetylcholine and nitroglycerin (B).

The distribution of vessel diameters in SDF and IDF image formats was analyzed and compared (Figure 2). The increase in pixel density and FOV did not influence this distribution. In the physiological experiment, more capillaries were detected at baseline in the IDF format, resulting in a higher TVD. However, the microcirculatory response to pharmacological stimulation with nitroglycerin led to a comparable increase in TVD in both SDF and IDF formats (*p* < 0.001). These findings suggest that the improvements in pixel density and FOV do not alter the observed microcirculatory changes in response to pharmacological stress in the SDF image format.

### 3.2 RBC Velocities

The use of the IDF image format revealed lower RBCv values compared to the SDF image format and the two alternative stabilization algorithms, AVA and CCTools (*p* < 0.0001). This difference may be attributed to the higher resolution of the IDF format, which allows for a more precise analysis of very slowly moving packed RBCs in venules. At lower resolution, such RBCs were previously classified as non‐moving (plugged vessels).

Notably, fewer IDF image format sequences were excluded based on quality criteria. The IDF image format demonstrated greater robustness against poor quality, consistent with the microcirculation image quality score [10, 20] and the second consensus on the assessment of sublingual microcirculation in critically ill patients [4].

### 3.3 Stabilization

Table 3 compares the performance of different stabilization algorithms in terms of correlations and levels of agreement across various microcirculatory parameters. The MicroTools algorithm was evaluated alongside the AVA and CCTools algorithms. Results demonstrated that all three algorithms were comparable for TVD, FCD, and PPV parameters, showing a good level of agreement, minimal bias, and strong correlation, particularly when compared to the AVA algorithm.

**TABLE 3:** Functional parameters of the sublingual microcirculation as measured with the different stabilization algorithms.

	AVA 3.2 stabilization algorithm	MicroTools 65 stabilization algorithm	CCTools 1.7.12 stabilization algorithm	Correlation analysis	*p*_corr_	*r*	Upper LOA	Lower LOA	Bias	Precision	Percentage error
	*n* = 178	*n* = 120	*n* = 188								
Image properties											
Field of view [mm^2^]	0.49 ± 0.04	0.48 ± 0.05	0.59 ± 0.01								
Pixel density [μm px^−1^]	1.21 ± 0.01	1.21 ± 0.01	1.21 ± 0.00								
Horizontal pixels [px]	661 ± 31	654 ± 37	716 ± 6								
Vertical pixels [px]	512 ± 31	505 ± 39	570 ± 12								
Microcirculatory parameters											
Small vessels (capillary) TVD [mm mm^−2^]	17.1 ± 3.6	17.8 ± 4.0	17.8 ± 5.4	AVA‐CCT	< 0.0001	0.74	4.5	−7.9	−1.7	3.1	−7
				MT‐CCT	< 0.0001	0.69	2.3	−9.8	−3.7	3	−16.8
FCD [mm mm^−2^]	15.5 ± 3.7	16.5 ± 3.9	16.6 ± 5.1	AVA‐CCT	< 0.0001	0.73	4.5	−7.7	−1.6	3.1	−6.8
				MT‐CCT	< 0.0001	0.70	2.1	−9.9	−3.9	3	−19.2
PPV [%]	91 ± 1	93 ± 8	94 ± 7	AVA‐CCT	< 0.0001	0.78	0.1	−0.1	0	0	0.1
				MT‐CCT	< 0.0001	0.76	0.1	−0.2	0	0.1	−3
RBCv [μm s^−1^]	336 ± 72	321 ± 71	337 ± 72	AVA‐CCT	< 0.0001	0.90	50.4	−71.4	−10.5	30.5	−3.2
				MT‐CCT	< 0.0001	0.90	66.8	−65.4	0.7	33.1	0
Large vessels TVD [mm mm^−2^]	7.1 ± 2.0	7.1 ± 2.1	7.2 ± 1.8	AVA‐CCT	< 0.0001	0.64	3.2	−3.6	−0.2	1.7	−0.6
				MT‐CCT	< 0.0001	0.62	2.9	−3.7	−0.4	1.7	−2.8

*Note:* Values are given as mean ± SD.

Abbreviations: FCD, functional vessel density; LOA, level of agreement; PPV, proportion of perfused vessels; RBCv, red blood cell velocity; TVD, total vessel density.

The FOV was larger with the CCTools stabilization algorithm compared to AVA and MT. However, unlike MicroTools, which cropped the image to the common data window shared across all frames, CCTools retained non‐moving (black) areas within the image. This inclusion resulted in an artificially enlarged FOV, as the non‐moving data could not be analyzed. Consequently, not all image data in the CCTools output was available for meaningful analysis.

## 4 Discussion

In this study, we demonstrated that full‐frame analysis using MicroTools increased the FOV by 200% and improved accuracy, as shown by a reduction in the standard deviation of each analyzed microcirculatory parameter. Additionally, a 66% increase in pixel density enhanced capillary detection, resulting in higher TVD. The microcirculatory response to a pharmacological challenge was found to be consistent between full‐frame analysis (IDF format) and SDF image formats. In summary, the full‐frame analysis provided by the Cytocam‐IDF in IDF image format enabled a more refined and accurate assessment with MicroTools, demonstrating good‐to‐excellent agreement with standard software (AVA). Furthermore, the stabilization algorithm implemented in MicroTools was as effective as the CCTools and AVA algorithms.

### 4.1 Analysis of High‐Resolution Handheld Vital Microscopy Image Sequences

High resolution handheld vital microscopy has been in regular use in research since 2013 [3, 9, 21], offering a fourfold increase in the number of pixels per frame compared to SDF imaging and improving the signal‐to‐noise ratio through 1:4 pixel binning in a high‐definition complementary metal‐oxide‐semiconductor (CMOS) image sensor [3]. The level of contrast and brightness of the image is markedly different between IDF and SDF and cannot be adjusted by altering the exposure settings because the inherent optical design and illumination principle of IDF microscopy intrinsically yield higher contrast and sharper vessel edges. Previous studies have shown that SDF imaging provides increased capillary contrast and image quality compared to the older OPS devices, with venular granularity being more clearly observable [2]. In a study comparing SDF and IDF imaging devices for assessing cutaneous microcirculation in preterm neonates, IDF imaging visualized 20% more vessels (TVD 16.9 vs. 14.1 mm.mm^−2^, *p* < 0.001) [22]. Aykut et al. demonstrated that Cytocam‐IDF provided improved image quality in terms of contrast and sharpness, with IDF images recorded using Cytocam‐IDF showing a 30% higher TVD compared to SDF images recorded with older SDF cameras [3]. Furthermore, Gilbert‐Kawai et al. showed that sublingual microcirculatory image acquisition, as assessed using the microcirculatory image quality scoring system, was superior with the Cytocam‐IDF microscope compared to the SDF video microscope, particularly for stability, pressure, and content parameters [23]. However, due to limitations in “gold‐standard” manual analysis software, all image sequences were down‐sampled to SDF resolution and FOV before analysis, resulting in significant data loss. Thus, a significant amount of data is lost and therefore not analyzed. A comparison of the MicroScan USB3 with the MicroScan analogue, both relying on SDF imaging, revealed superior image quality with the USB3, particularly in terms of illumination, focus, and pressure optimization [24]. To date, no direct comparison has been performed between Cytocam‐IDF and MicroScan USB3; only comparisons between Cytocam‐IDF and the older MicroScan analogue are available [6].

During the conversion from IDF to SDF image format, both the FOV and resolution (pixel density) are downgraded. The FOV can vary between individual image sequence clips due to the stabilization process, as more unstable (moving) video clips result in greater data loss and a reduced FOV. In practice, full‐frame analysis using the IDF image format led to a 10% increase in TVD in addition to the 30% improvement already achieved with the transition from the SDF to the IDF imaging device.

In the present study, the total vessel density (TVD) for venules (large vessels) also increased, albeit to a lesser extent than for capillaries. This is expected, as venules are less likely to be missed in the lower‐resolution SDF image format files. However, due to their less frequent occurrence, venules are more susceptible to selection bias caused by the field of view (FOV). This bias is mitigated by the larger FOV provided by full‐frame analysis. Consequently, the increase in venular TVD is likely attributed to the expanded FOV, whereas the increase in TVD for capillaries is primarily due to the higher pixel density (μm/px resolution).

Gilbert‐Kawai et al. studied the changes in labial capillary density during the Xtreme Everest 2 research expedition [25]. In their study, the authors used full‐frame analysis with the Cytocam‐IDF device; however, video analysis was performed using only CCTools v1, relying on manual counting of labial capillary loops. As such, the study did not permit any meaningful comparison with AVA software or MicroTools.

Another intravital microscope, the MicroSee V100 (Guangzhou Medsoft System LTD, Guangzhou, China), has been previously used [26, 27, 28]. However, its technical specifications are scarcely available, and no study to date has compared the MicroSee to other handheld vital microscopes. The MicroSee utilizes blue light LEDs. In healthy volunteers, blue light imaging resulted in significantly higher perfused vessel density (mean increase of 4.6 ± 4.7 mm.mm^−2^, *p* < 0.0001) and total vessel density (mean increase of 5.1 ± 4.6 mm.mm^−2^, *p* < 0.0001) compared to green light. Additionally, the blue light probe yielded a significantly lower rate of poor‐quality recordings than the green light probe (10% vs. 39%, *p* < 0.0001). Overall, blue light provided superior microcirculatory vessel density and image quality compared to the green‐light SDF probe without increased FOV [28].

### 4.2 Effects of High‐Resolution Image Sequence Analysis on Quantification of RBCv

To date, this is the first study to analyze RBCv using the full‐frame IDF image format. A lower venular RBCv was observed in full‐frame analysis compared to the SDF image format. This can be explained by the inability, at lower resolution, to discern tightly packed RBCs in venules. By contrast, the higher resolution provided by the IDF format allowed slow RBCs' movements to be detected, resulting in a lower overall RBCv. This enhanced resolution is not only valuable for RBCv analysis but also facilitates the identification and tracking of leukocytes—whether rolling or stationary—within the postcapillary venules.

### 4.3 Automated Image Sequence Stabilization

Automated analysis of HVM image sequences is more sensitive to imperfect stabilization than manual methods. In AVA software, stabilization includes optional image enhancement steps. First, intensity variations in each frame's background are reduced by subtracting the best‐fitting quadratic polynomial surface and then adding the original image's average intensity. Second, contrast is improved by adjusting the image gray‐scale histogram through a transfer function that maps each input gray level to a new output gray level.

Our findings indicate that using two independently developed, modern stabilization algorithms produced algorithmic analysis results that were successfully validated against manual analysis. In contrast, applying a stabilization algorithm originally intended for manual analysis led to less accurate algorithmic performance [7, 8]. Thus, the MicroTools image sequence stabilization algorithm meets the stringent requirements needed to fully leverage automated and real‐time image sequence analysis [29, 30].

## 5 Limitations

Our study has several limitations. First, we did not conduct a mathematical comparison of the algorithms themselves, but rather compared the microcirculatory parameters derived from each analysis method. Nonetheless, previous studies have employed similar approaches to compare software and microcirculatory parameters [29, 30]. Second, the AVA 3.2 software can only stabilize image sequences in the SDF image format. As a result, the sequences used for MicroTools stabilization were also in SDF format. Third, we did not validate the increase in capillary detection with an enlarged field of view in critically ill patients or other clinical contexts. Such validation will be addressed in a larger study, as it requires specific authorizations that are not yet available, and because video acquisition quality in critically ill patients is inherently more variable than in controlled volunteer settings.

## 6 Conclusion

Full‐frame analysis (IDF image format) of Cytocam IDF images using MicroTools is both feasible and significantly enhances the field of view while improving accuracy. By utilizing the full IDF image format, more capillaries are detected, resulting in a higher total vessel density. MicroTools demonstrated notable advantages compared to other analysis software. These findings suggest that full‐frame analysis should become standard practice when using high‐resolution Cytocam IDF devices and hold substantial potential for advancing research into the microcirculation under both healthy and diseased conditions.

## 7 Perspectives

New analytical standard with full‐frame IDF imaging for microcirculatory analysis: Full‐frame IDF imaging significantly improves capillary detection, supporting its adoption as the new standard in microcirculatory analysis.

Robust automation with fully integrated stabilization and analysis pipeline: The integrated MicroTools algorithm enables accurate, automated, and device‐independent image stabilization and analysis.

Clinical relevance: Enhanced precision in microvascular metrics advances real‐time, bedside assessment of microcirculatory function in critical care.

## Author Contributions

M.P.H. designed the study, developed the algorithms described in this manuscript and wrote the software implementation, performed data analysis, wrote the manuscript. P.G. designed the study, wrote the manuscript, edited the manuscript. T.B., C.L., J.M., and O.D. wrote the manuscript, edited the manuscript; C.I. designed the study, edited the manuscript. All the authors approved the final manuscript.

## Funding

This research was supported in part by a University of Zurich Walter und Gertrud Siegenthaler Foundation grant issued to M.P.H.

## Ethics Statement

Informed consent for publication of anonymized data has been obtained from each subject prior to enrollment, as mandated by the Declaration of Helsinki and in accordance with the institutional Ethics Board of the University of Bern (KEK 226/12, http://clinicaltrials.gov identifier NCT01953198).

## Conflicts of Interest

Can Ince who is the chief scientific officer of Active Medical BV, Leiden, The Netherlands, a company that provides devices, software, education, and services related to clinical microcirculation. Matthias Hilty is a shareholder of Active Medical BV (Leiden, the Netherlands). The other authors declare no conflicts of interest.

## Supporting information


**Data S1:** micc70045‐sup‐0001‐supinfo.docx.

**Figure S1:** Digital image processing workflow.

## Data Availability

An archive of the original source code (git repository) for Microtools is available in the Zenodo repository under the GPLv3 open‐source license (https://zenodo.org/records/2608759). The algorithm configuration parameters used to process the clinical data in this article are available in the Zenodo repository. All data is available upon reasonable request.
