# Supplementary material for: Improved Detection of Capillaries in High‐Resolution Handheld Vital Microscopy by Use of the MicroTools Advanced Computer Vision Algorithm
Source: Microcirculation. 2026 Jan 4;33(1):e70045. doi: 10.1111/micc.70045 (PMC12765483; doi:10.1111/micc.70045)
Supplement: Supplementary file 1 — Data S1: micc70045‐sup‐0001‐supinfo.docx. [file MICC-33-e70045-s001.docx]

# Microcirculation Image Ǫuality Score


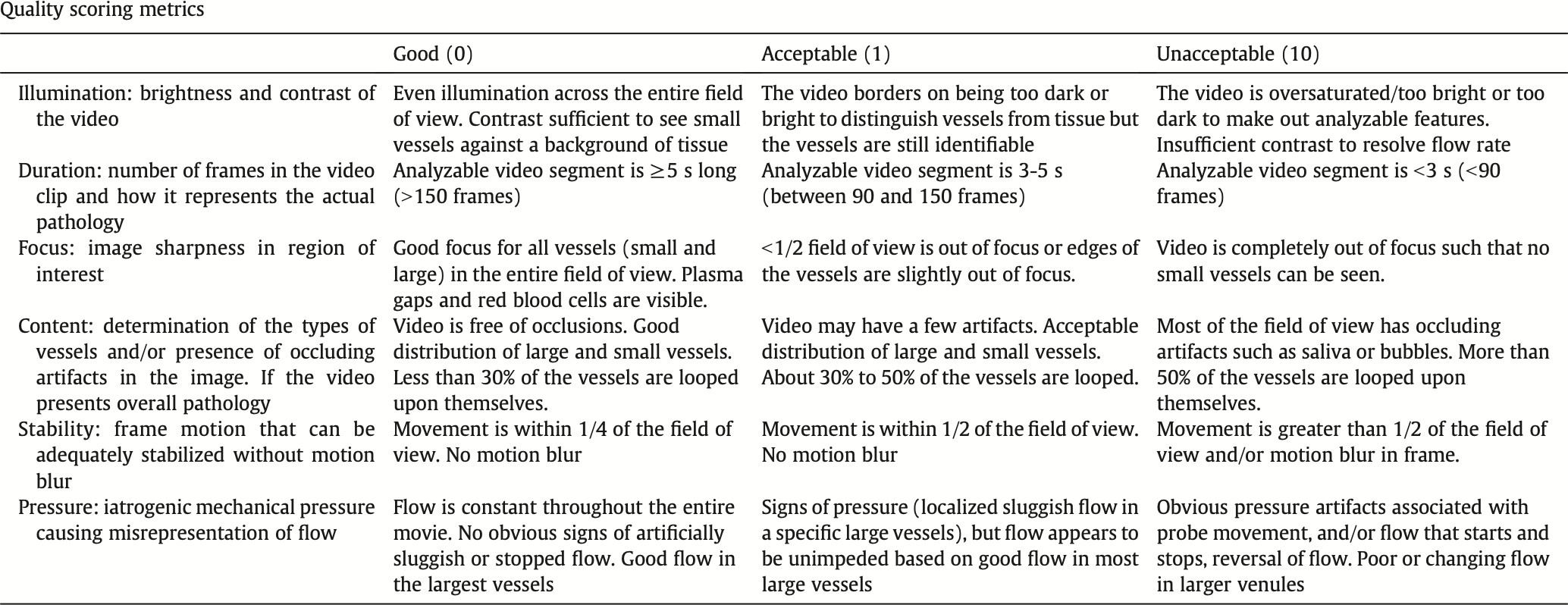


From Massey et al. [1]

[1] Massey MJ, LaRochelle E, Najarro G, Karmacharla A, Arnold R, Trzeciak S, Angus DC, Shapiro NI. The microcirculation image quality score: Development and preliminary evaluation of a proposed approach to grading quality of image acquisition for bedside videomicroscopy. J Crit Care 2013;28:913–7. https://doi.org/10.1016/j.jcrc.2013.06.015.

# Supplementary Figure

Supplementary Figure 1. Digital image processing workflow

**Conversion by CCT**

n = 324

HVM image sequences successfully stabilized by CCT in IDF FOV and resolution

n = 286

HVM image sequences successfully converted to SDF FOV and resolution

**Stabilization by CCT**

n = 4 stabilization rejected in CCT

n = 42 conversion failed

due to file corruption

n = 328

HVM image sequences

recorded

in IDF FOV and resolution

n = 6 conversion failed n = 29 stabilization

rejected in AVA

n = 2 stabilization rejected in MT

**Conversion by CCT**

**Stabilization by AVA**

**Stabilization by MT**

n = 318

HVM image sequences successfully converted to SDF FOV and resolution

n = 257

HVM image sequences successfully stabilized by AVA in SDF FOV and resolution

n = 284

HVM image sequences successfully stabilized by MT in SDF FOV and resolution

matched to both

groups

n = 5, n = 82 stabilization

/ quality criteria rejected n = 98, n = 108, n = 166

stabilization

/ quality criteria rejected

**Analysis by MT**

**Analysis by MT**

**Analysis by MT**

**Analysis by MT**

**Analysis by MT**

n = 323

HVM image sequences analyzed by MT and passed quality criteria

in IDF FOV and resolution

n = 242

HVM image sequences analyzed by MT and passed quality criteria

in SDF FOV and resolution

n = 188

HVM image sequences analyzed by MT and passed quality criteria

in SDF FOV and resolution

n = 178

HVM image sequences analyzed by MT and passed quality criteria

in SDF FOV and resolution

n = 120

HVM image sequences analyzed by MT and passed quality criteria

in SDF FOV and resolution

Comparison of stabilization algorithms

Comparison of field of view and pixel pitch
