# Supplementary figures and images for: Improved Detection of Capillaries in High‐Resolution Handheld Vital Microscopy by Use of the MicroTools Advanced Computer Vision Algorithm
Source: Microcirculation. 2026 Jan 4;33(1):e70045. doi: 10.1111/micc.70045 (PMC12765483; doi:10.1111/micc.70045)

# Supplementary figures

Supplementary Figure 1. Digital image processing workflow.


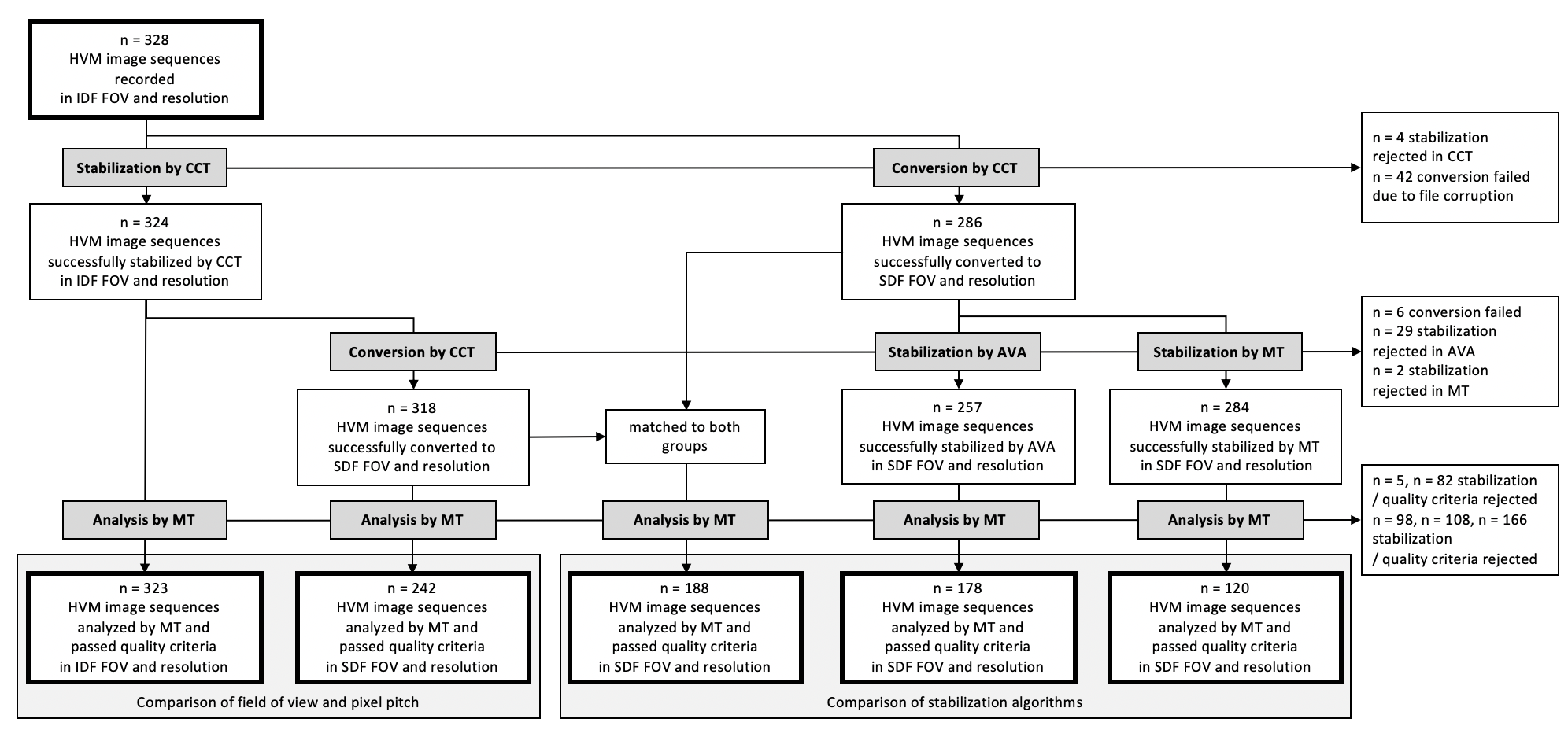

Supplement: Supplementary file 2 — Figure S1: Digital image processing workflow. [file MICC-33-e70045-s002.docx]
